# The Active for Life Year 5 (AFLY5) school based cluster randomised controlled trial: study protocol for a randomized controlled trial

**DOI:** 10.1186/1745-6215-12-181

**Published:** 2011-07-24

**Authors:** Debbie A Lawlor, Russell Jago, Sian M Noble, Catherine R Chittleborough, Rona Campbell, Julie Mytton, Laura D Howe, Tim J Peters, Ruth R Kipping

**Affiliations:** 1 MRC Centre for Causal Analyses in Translational Epidemiology, University of Bristol, UK; 2 School of Social & Community Medicine, University of Bristol, UK; 3 Centre for Exercise, Nutrition & Health, School for Policy Studies, University of Bristol, UK; 4 Discipline of Public Health, School of Population Health and Clinical Practice, University of Adelaide, UK; 5 Department of Health and Applied Social Sciences, University of West of England; 6 Public Health Directorate, NHS Bristol, UK; 7 School of Clinical Sciences, University of Bristol, UK

## Abstract

**Background:**

Low levels of physical activity, high levels of sedentary behaviour and low levels of fruit and vegetable consumption are common in children and are associated with adverse health outcomes. The aim of this paper is to describe the protocol for a cluster randomised controlled trial (RCT) designed to evaluate a school-based intervention that aims to increase levels of physical activity, decrease sedentary behaviour and increase consumption of fruit and vegetables in school children.

**Methods/design:**

The Active for Life Year 5 (AFLY5) study is a school-based, cluster RCT that targets school children in Year 5 (age 9-10 years). All state junior/primary schools in the area covered by Bristol City and North Somerset Council are invited to participate; special schools are excluded. Eligible schools are randomised to one of two arms: intervention arm (receive the intervention 2011-2012) and control arm (receive the intervention after the final follow-up assessment, 2013-2014). The primary outcomes of the trial are levels of accelerometer assessed physical activity and sedentary behaviour and questionnaire assessed fruit and vegetable consumption. A number of secondary outcomes will also be measured, including body mass index, waist circumference and overweight/obesity. Outcomes will be assessed at baseline (prior to intervention when the children are in Year 4), at the end of intervention 'immediate follow-up' and '12 months long-term' follow-up. We will use random effects linear and logistic regression models to compare outcomes by randomised arm. The economic evaluation from a societal perspective will take the form of a cost consequence analysis. Data from focus groups and interviews with pupils, parents and teachers will be used to increase understanding of how the intervention has any effect and is integrated into normal school activity.

**Discussion:**

The results of the trial will provide information about the public health effectiveness of a school-based intervention aimed at improving levels of physical activity, sedentary behaviour and diet in children.

**Trial registration:**

ISRCTN50133740

## Background

Low levels of physical activity, high levels of sedentary behaviour and low levels of fruit and vegetable consumption, all of which are associated with adverse health outcomes, are common in children in the UK and other high income countries [1-7]. Since almost all children attend school, school-based interventions have the potential to efficiently change behaviours to be more health promoting. However, current evidence for the effectiveness of school-based interventions is limited by important sources of bias.

A Cochrane systematic review of school-based physical activity programmes in children and adolescents identified 26 relevant studies (15 randomised controlled trials (RCTs) and 11 quasi-experimental studies) [8]. The majority (16/26) of studies were from the US, with none from the UK. In all but one study the intervention included curricular-based activities that provided physical activity during school hours. Of nine outcomes assessed, school-based physical activity interventions showed a beneficial effect on four: duration of physical activity (achieved largely by increased activity in school), television viewing, cardio-respiratory fitness and blood cholesterol [8]. Two key weaknesses of most studies included in the review were noted. First, the vast majority used self- or parental-report of physical activity and sedentary behaviours and there was some evidence that the use of self-report biased results towards a more beneficial impact of the intervention [8]. Second, all but one study examined outcomes only at the end of the intervention. This is an important weakness since a key effect was likely to be the direct result of increased class-based physical activity, which to some extent the children are compelled to do. Whilst this is likely to be beneficial in the short term it is also important to know whether healthy behaviours continue beyond the period of the intervention.

A systematic review of controlled studies with interventions to reduce sedentary behaviour identified 12 studies (11 RCTs and 1 quasi-experimental); 6 were in clinical obese populations and 6 were general population prevention studies [9]. All of the general population prevention studies were school-based, of which 5 were curriculum-based with health promotion activity lessons; one was extracurricular only (after school dance classes and home visits). In all 6 studies screen viewing time declined markedly in children from the intervention schools (decline ranging from 43% to 16%) and either decreased only slightly or increased in the control groups (ranging from 14% decline to 12% increase) [9]. Outcomes were only assessed at the end of the intervention and parental/child report of sedentary behaviour was used for the key outcome measure in all six studies.

At least ten published systematic reviews of school-based intervention studies aimed at preventing childhood overweight/obesity have been published [10]. Differences in the reviews include the time periods of the reviews, the inclusion and exclusion criteria, whether meta-analysis was undertaken and how outcomes were assessed. Two included meta-analyses and these both reported a protective effect of school-based interventions: odds ratio of overweight or obesity of 0.74; 95% CI: 0.60 to 0.92[11] and a standardised mean difference in weight of -0.29; 95% CI: -0.45 to -0.14 [12]. There is some evidence that the quality of trials has improved over time [10]. However, the vast majority of trials are from the US, most assessed outcomes only at the end of the intervention and few used objective measurements for assessing changes in physical activity and sedentary behaviour [10].

We have completed feasibility and pilot work of a school-based intervention - Active for Life Year 5 (AFLY5) - which is aimed at increasing physical activity, reducing sedentary behaviour and increasing consumption of fruit and vegetables in 9-10 year olds [13,14]. The intervention is adapted for the UK school context from the Planet Health and Eat Well Keep Moving intervention from the US [15]. Our feasibility and pilot work demonstrates that AFLY5 is acceptable and feasible to deliver, and appears to have beneficial effects on physical activity, screen viewing and fruit and vegetable consumption [13,14].

Although the underlying causes for the pattern are unknown, recent evidence suggests that most childhood weight gain occurs in mid-childhood (age 7-11 years) in UK children [16,17]. If further research confirms that changes in diet, physical activity and sedentary behaviour around this age are related to this increase in weight gain the AFLY5 intervention (aimed at 9 to 10 year olds) may be particularly important to improving public health should it turn out to be effective in generating healthy changes in these behaviours.

### Aims

The aims of AFLY5 are to determine the effectiveness and cost-effectiveness of the intervention in children aged 9-10 years to improve the following primary outcomes:

1. Daily time spent in, and amount of, physical activity.

2. Daily time spent in sedentary behaviour.

3. Portions of fruit and vegetables consumed per day.

And secondary outcomes:

1. Time spent screen-viewing per day.

2. Portions of: snacks; high fat foods; and high energy drinks consumed per day.

3. Body mass index (BMI).

4. Waist circumference (WC).

5. Whether overweight/obese.

The aim is to determine whether the intervention affects these outcomes in the short-term (i.e. immediately at the end of the intervention) and in the longer term (i.e. 12 months after the end of the intervention).

## Methods/design

AFLY5 is a school-based, cluster RCT.

### Recruitment of participants and baseline assessment

**Figure 1:**
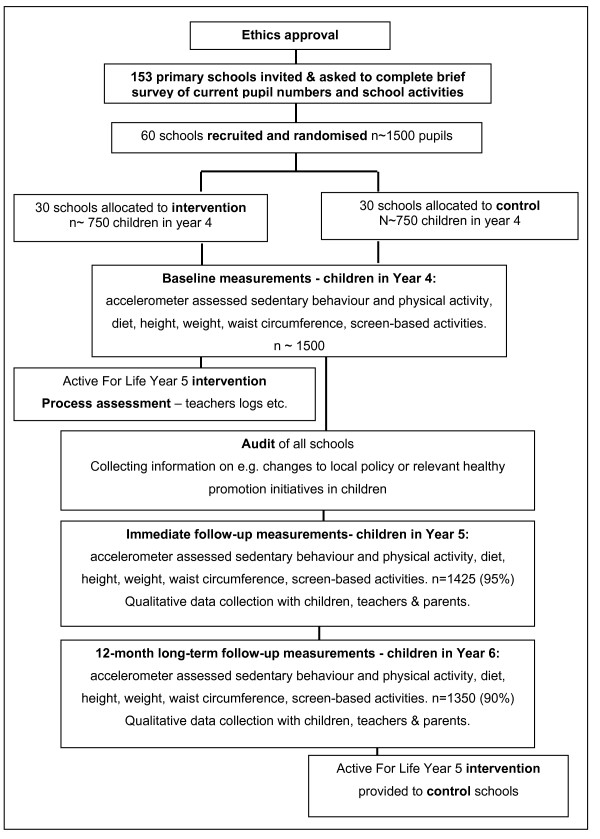
**Planned flow of participants through the Active for Life Year Five school-based randomised controlled trial**.

All state primary and junior schools with children in Years 4-6 (aged 8-11) in the area covered by Bristol City Council and North Somerset Council will be invited to participate. Both of these areas are in the South West of England and include a range of levels of deprivation, as well as urban and rural areas. Special schools (that is, those for children whose additional needs cannot be met in a mainstream setting) will be excluded because they are unlikely to be teaching the standard national curriculum and the children may not be able to take part in all the measurements. Participants will be children in Year 4 at the recruitment stage. Baseline assessment (prior to intervention) will be undertaken when these children are in the final terms of Year 4. The intervention will take place when the children are in Year 5. Figure 1 shows the planned flow of participants through the study.

### Randomisation procedure

Prior to inviting schools to participate we will identify all local and national initiatives that currently target primary/junior schools and are aimed at increasing physical activity, decreasing sedentary behaviour and improving diet. We will include a brief questionnaire with the letter of invitation to schools requesting information on involvement of the school in any initiatives to change the children's behaviour that our intervention is aimed at influencing. We will use this information to define recruited schools as high or low involvement in current initiatives. We will define recruited schools as high, mid or low-deprivation by thirds of their score on the Index of Multiple Deprivation 2010 (IMD 2010) [18]. We will group schools by level of involvement in current initiatives and thirds of deprivation score (total of 6 groups/strata) and randomly allocate them within these strata to control or intervention. Randomisation will be undertaken by a researcher unaware of any characteristics of the schools and will be concealed by using the Bristol Randomised Trials Collaboration's automated (remote) system.

### Follow-up

Children will be assessed on three occasions, planned to take place at the following times:

1. Baseline, between May and October 2011, when the children are in school Year 4 or the first term of Year 5.

2. Immediate follow-up, between April and September 2012, when the children are largely in school Year 5 (some assessed in the first month of Year 6) and at the very end of the intervention.

3. 12 months long-term follow-up, between March and July 2013, when the children are in school Year 6.

#### Methods for dealing with loss to follow-up

The school years for all of our assessments are in primary/junior school and data from Bristol City Council shows very little (~5% of children) movement of children between schools or leaving school in the Bristol area over these school years; where movement does occur it tends to be to schools in neighbouring city council areas, such as North Somerset. We had very few problems during the pilot and feasibility studies, with just 2% of participants leaving their school and 8% missing one or other of the baseline or follow-up assessments because of school absenteeism. In this full scale trial fieldwork will be organised so that children who are out of school (for example, due to illness) on the day of data collection will have the opportunity to provide some data on an alternative day, which was not possible in the pilot/feasibility work. We will work closely with schools to ensure that visits for any of the assessments do not coincide with school trips or other activities that would make data collection difficult. Our sample size calculation anticipates a 15% loss to follow-up by the 12 month long term follow-up, which experience leads us to believe is likely to be an overestimate.

### Intervention

Schools randomised to the intervention group will receive the intervention immediately and those randomised to control group after the completion of the final 12 month long term follow-up assessment. The intervention comprises:

1. Training for Year 5 classroom teachers.

2. Provision of 16 lesson-plans and teaching materials, including pictures, CDs and journals.

3. Provision of 10 parental-child interaction homework activities.

4. Information in the school newsletters about the importance of increasing physical activity, reducing sedentary behaviour and improving diet.

5. Written information for parents on how to encourage their children to eat healthily and be active.

The intervention was adapted from a previously evaluated US intervention[15] and is based on Social Cognitive Theory,[19] with a particular emphasis on increasing the children's self-efficacy (perceived competence) to be physically active and eat a healthy diet [20]. Training for classroom teachers takes one day and will be provided by the trial manager and two local teachers. We will offer a choice of one of two training days (aiming for ~ 15 teachers at each day), but will provide additional days if these are not suitable for some teachers. Costs of a supply teacher will be provided as part of the intervention (this worked without problems in the feasibility/pilot studies). At the end of the training the teachers will be provided with lesson plans and all necessary materials for the 16 lessons and 10 homeworks, as well as contact details of the trial manager. The lessons and homeworks are summarised in Table 1. Each lesson covers specific topics but there is overlap to reinforce messages from other lessons. The intervention will be delivered between the beginning of September 2011 and end of April 2012 for those schools where the baseline assessment is completed before the end of the Year 4 school year (i.e. by 22^nd ^July 2011) and between mid-November 2011 and end of September 2012 for those schools where the baseline assessment is completed in the first term of Year 5.

**Table 1:** Lesson Titles and Learning Objectives

Title	Summary of learning objective	Homework
Fit Check 1	Introduce students to keeping a record of physical activity	**Goal setting**: increasing activity and reducing TV. Scavenger hunt list included as suggestion.

Fit Check 2	Introduce children to interpreting results and setting goals	

Safe workout: PA Introduction (theory)	Identify and sequence the components of a safe and healthy work out Demonstrate a safe work out	

Balance of Good Health (nutrition)	Understand concept of healthy living Balance of Good Health and its importance for a healthy diet	**Cooking at home: **two recipes

Five foods countdown (PA)	Complete an endurance work out Move for a set time without stopping List a variety of foods from the food groups Demonstrate awareness of five food groups in Balance of Good Health	

Five food groups (nutrition)	Role of different nutrients (especially macronutrients) Serving recommendations and portion sizes	**Blank Eat Well Plate: **complete all food eaten in one day by food group

Musical Fare (PA)	Demonstrate and complete an endurance activity to music Demonstrate a pace that works for a set time (using music & dance) Teach knowledge of the five food group in Balance of Good Health (integrated with PA exercise)	

Keeping the balance (nutrition)	Meaning of balance Importance of a balanced diet	**Bingo challenge card**: choice of 10 activities to do out of 40

Three kinds of fitness (PA)	Demonstrate five parts of a safe workout Demonstrate different exercises that help improve endurance, strength and flexibility fitness Identify different parts of fitness	

Freeze my TV (sedentary behaviour)	Analyse leisure time to identify time spent watching TV Create list of alternative activities	**Freeze My TV**: a family game aimed at switching the TV off at planned times and doing an alternative activity together

Snack attack (nutrition)	Describe the importance of selecting healthy snacks Analyse food labels to locate nutritional information and fat content	**Snack worksheet**: comparing food content of two snacks at home

Bowling for snacks (PA)	Demonstrate an endurance workout Demonstrate a pace that they can follow for a set time Describe a healthy snack (integrated with PA exercise) Categorise a healthy snack (integrated with PA exercise)	**Bowling for snacks**: a game played with parents that involves identifying healthy snacks and replacing unhealthy snacks with healthy ones.

Think about your drink	Measure the amount of sugar consumed from soft drinks and evaluate the results. Learn to replace soft drinks and other sugar-sweetened beverages with healthy drinks like milk and water.	**Sugar in drinks: **instructions for calculating and measuring sugar in drinks at home

Veggiemania (PA)	Complete an endurance workout Demonstrate a pace that works for a set time Learn the importance of eating five fruit and vegetables a day (integrated with PA exercise)	**5 A Day: **weekly planning sheet of ideas for increasing fruit and veg. consumption and checking whether these are achieved

Brilliant Breakfast (nutrition)	Knowledge of the importance of having a healthy breakfast Knowledge of the consequences of not having a healthy breakfast	**Brilliant breakfast**: writing a letter to a character who does not normally eat breakfast. The brief letter should persuade them to eat a healthy breakfast.

Fit Check	Revisit and redo the Fit Check.	

PA: Physical activity class

### Outcome measurements

The **primary outcomes **are:

1. Accelerometer assessed mean time per day spent doing moderate/vigorous physical activity (MVPA).

2. Accelerometer assessed mean time per day spent in sedentary activity.

3. Self-reported (validated questionnaire) portions of fruit and vegetables consumed per day.

The **secondary outcomes **are:

1. Self-reported (validated questionnaire) mean time spent screen-viewing.

2. Self-reported (validated questionnaire) portions of: snacks; high fat foods; and high energy drinks consumed per day.

3. BMI determined from weight and height measured in classrooms by two study fieldworkers.

4. WC measured in classrooms by two study fieldworkers.

5. Overweight/obesity, determined by appropriate gender and age specific thresholds from BMI and WC growth charts.

We do not see the timing of follow-up (immediate or 12 month long-term follow-up) in terms of primary or secondary outcomes, but rather as having equal importance in assessing the primary and secondary outcomes (as above) at these two different time points.

A cost consequence analysis will be conducted in which the costs to society of the intervention will be compared with both primary and secondary outcomes.

The potential **mediators **(based on Social Cognitive Theory [19]), that we will assess among children are:

1. Physical activity and sedentary behaviour self-efficacy.

2. Fruit and vegetable consumption self-efficacy.

3. Report of parental support for physical activity.

4. Report of parental support for consuming healthy levels of fruit and vegetables.

Assessments will be undertaken by trained fieldworkers who will have completed enhanced criminal records bureau (CRB) checks as required for those working with children in the UK. The fieldworkers will not be told which schools have been allocated to which arm of the trial. At each stage of assessment (baseline, immediate- and 12 month long-term follow-up) the following will be measured on the children in both intervention and control schools:

#### Accelerometer assessment of physical activity and sedentary behaviour

We will use ActiGraph accelerometers[21] and the same protocol that we used in our feasibility/pilot work. Accelerometers will be set to record at 10-second epochs on the day after the accelerometers are handed out. Where possible, accelerometers will be given out on a Wednesday and will be collected the following Tuesday, downloaded, recharged, reinitialised and taken to the next school the following day. The accelerometers will be shown to the children and verbal instructions provided in the class room with all children together. Each child will be given their accelerometer individually at the time of the anthropometric measurements and the child will be asked what they remembered about when the accelerometer should be worn and removed. They will be asked to wear them during the day (except when bathing or swimming or participating in contact sports such as karate) until the fieldworkers return to collect them. This will allow data collection on three weekdays (Thursday, Friday and Monday) and both weekend days. We will use established procedures for analysing the accelerometer data and calculate time spent in MVPA and sedentary behaviour.

#### Weight, height and waist circumference

All anthropometric measurements will be completed with children in a private room with both CRB checked trained fieldworkers present. Weight will be measured without shoes in light clothing to the nearest 0.1 kg using a Seca digital scale. Height will be measured, to the nearest 0.1 cm, without shoes using a portable Harpenden stadiometer. Fieldworkers will be trained to ensure correct position for height assessment. WC will be measured the nearest 1 mm at the mid-point between the lower ribs and the pelvic bone[22] with a flexible tape. BMI and WC will be used to define whether a child is generally overweight/obese (using BMI) or centrally overweight or obese (WC), using appropriate gender and age specific thresholds from BMI and WC growth charts [23-25]. For BMI we will use both international obesity task force thresholds and those based on the UK 1990 growth charts[23] and will compare results using these two different definitions.

#### Questionnaire assessment of diet, physical activity and sedentary behaviour

Fruit and vegetable consumption and other dietary outcomes will be assessed using the "A Day in the Life Questionnaire" (DILQ),[26] which was used in our feasibility/pilot studies [14]. The DILQ provides information about the children's entire food and drink intake the previous day. To improve recall the questionnaire is structured with sequential questions in a 24 hour segmented school day. Fruit and vegetable consumption and other dietary outcomes will be assessed using an established scoring scheme [14,27,28]. As in the pilot study, allocation of foods written in text to categories will be undertaken independently by two individuals with discrepancies checked by a third independent individual, and a random 5% further coded by another researcher to check for accuracy. In the pilot/feasibility work there were very high levels of agreement (> 97%) [14]. An abbreviated and updated version of a previously validated screen viewing questionnaire was used to assess self-report sedentary behaviour in our feasibility/pilot study and this will be used here [29]. The questionnaire asks about the length of time spent doing screen based activities on the previous weekday and Saturday. The changes that we made (with the author's permission) included adding new media, such as Xbox and Play Station, and removing detailed questions about the number and location of TVs in the house and eating whilst watching TV, in order to reduce the length of the questionnaire.

#### Mediators assessment

We hypothesise that change in physical activity and fruit and vegetable consumption will be mediated by change in the child's self-efficacy. As the study is designed to increase parental support for physical activity we also hypothesise that parental support will function as a mediator of behaviour change. Increasing our understanding of the mechanisms of behaviour change is essential for designing and delivering more effective interventions and establishing how to disseminate a complex intervention that has been shown to be effective in one setting to another setting [30]. Therefore, we will assess four hypothesized mediators using well-established scales that have been shown to have good reliability among primary school aged children. Specifically, physical activity and screen-viewing self-efficacy will be assessed using the 9-item scale of activity and sedentary behaviour self-efficacy[31] and fruit and vegetable self-efficacy will be assessed using an 18-item scale that assesses child's self efficacy for this behaviour [32]. Parental support for physical activity will be assessed using the 18-item, child completed Activity support questionnaire which provides information on parental logistic support (that is, transport to activity venues, facilitating participation in organised activities), parental modelling of physical activity behaviours and parental support for reducing screen-viewing [33,34]. Perceived parental modelling for consuming fruit and vegetables will be assessed using a 15-item scale [35].

All questionnaires will be combined into one document and administered in the classroom (self-completed by the school children) with a fieldworker and teacher present to answer any queries and to assist the children with reading and writing as necessary. The fieldworkers and teachers will be instructed only to help with reading and spelling specific words and not to suggest answers to any questions.

#### Collection of information on resource use and costs

Resource use related to the administration of the intervention in the form of time, travel and materials will be collected through trainer and teacher time and resource logs completed over the intervention period. A parental completed postal questionnaire will be administered at the end of the intervention. The parents will be asked about their time spent in helping children with relevant homework, and taking children to and attending out of school activities, as well as any cost involved. They will also be asked about health service use in relation to exercise related injury in their child. To facilitate the completion of this questionnaire resource use logs, in which they can record this information, will be given to parents at the beginning of the year in which the intervention takes place. In order to compare costs between intervention and control schools the same questionnaire will be sent to parents at all schools.

#### Audits and process evaluation

Audits and process evaluations will be undertaken throughout the study to ascertain whether the intervention was delivered as planned and to identify any issues that might impact dissemination.

Audits will be undertaken at all schools, in both the intervention and control arms, to assess current physical activity provision (number of scheduled physical education (PE) lessons per week and time allocation for unsupervised outdoor play), school physical activity and nutrition polices (active travel, break-time play, packed lunch policy, etc) and number and type of after school clubs provided. Audits will be conducted once per term in all schools during the intervention and follow-up periods of the study (September 2011-July 2013).

We will ask all Year 5 teachers in intervention schools to complete a log of session delivery. The log will ask teachers to report the date that the sessions were delivered, the dates that homeworks were set and returned, and any problems or positive comments about the lesson.

Process evaluation will be conducted using focus groups with children, face-to-face interviews with teachers and school administrators, and telephone interviews with parents. Focus groups will be conducted at the end of the intervention in six purposefully selected intervention schools and six purposefully selected control schools with 6-8 purposefully selected pupils in each school. Schools will be selected to ensure representation of schools from more and less deprived areas and those with few and many programmes or initiatives (in addition to the study intervention) to promote healthy behaviours in children. Pupils will be selected to ensure both girls and boys are represented, as well as those who express strong and weaker self-efficacy and parental support at baseline assessment. The topic guides will be largely identical for both children in intervention and control groups. The focus groups will first examine the children's knowledge, attitudes and behaviours in relation to physical activity, sedentary behaviour and diet. They will then examine whether the children remember any lessons or homeworks that they have received during year 5 that relate to physical activity, sedentary behaviour and diet. If the children in intervention schools spontaneously mention intervention lessons and homeworks then the interviewer will probe about whether they liked or disliked these lessons (and ask for reasons) and what they remember learning from these lessons. Similar probing will take place with children in control schools where they spontaneously mention specific lessons or homeworks related to diet, exercise or sedentary behaviour topics. Children will be encouraged to say what they liked and disliked about these activity/diet related lessons and ideas they have about improving how these subjects could be taught. In any focus group (intervention or control school) where no specific lessons or homeworks related to physical activity, sedentary behaviour and diet are mentioned the pupils will be asked about whether they can describe any specific lessons/homeworks. In intervention schools only, the interviewer will describe some of the intervention lessons and homeworks to see if that prompts the children's memory of these if none have been spontaneously mentioned as the group appears to be coming to an end. If the children acknowledge some memory of these lessons/homeworks, they will then be prompted further about what they remember and what they liked or disliked about them. Thus, the focus groups will examine children's attitudes towards being taught these subjects and the methods of teaching them and will also assess whether intervention school children remember anything specific about the intervention and whether control school children received similar lessons.

All classroom teachers who delivered the intervention and head teachers (or their deputies) from all of the 25 intervention schools will be invited to participate in face-to-face, in-depth, semi-structured interviews that will assess aspects of the intervention that worked well, or that could be improved, with a focus on any elements that would improve delivery within the school. Finally, phone interviews will be conducted with a sample of 30 parents from 12 different schools (6 intervention and 6 control) to assess the parents' perspective of teaching their children about healthy levels of physical activity, sedentary behaviour and diet in school. The attitudes of parents from intervention and control schools towards parent involvement with homeworks will be explored, initially with no prompts (so that as with children we can explore whether parents remember the specific intervention homeworks and whether parents from control schools report any homeworks that appear similar to those from the intervention school). If parents from the intervention schools do not spontaneously mention any of the intervention homeworks they will be asked about specific homeworks. If they report remembering these, they will be asked about their attitudes towards the parent-child active homeworks that were part of the intervention.

### Statistical analyses

The presentation of findings from the trial will be in accordance with CONSORT guidelines for cluster RCTs [36]. Descriptive statistics (comparisons of means, medians or % as appropriate) will be used to assess any marked differences between intervention and control schools at baseline in terms of the outcome measurements, gender, age, school deprivation, participation of schools in other initiatives to promote healthy behaviours and neighbourhood characteristics.

The primary effectiveness analyses will be conducted on an intention to treat basis, with secondary (explanatory) analyses conducted first comparing outcomes in all control schools to outcomes only in those schools that completed at least 70% of the lessons, and secondly employing instrumental variables regression models to address the question of causal effect if the intervention were applied as intended [37]. For the instrumental variables regression analyses we will use the method proposed by Greenland for cluster RCTs [37]. Comparisons of means for the primary and secondary continuously measured outcomes will be presented as mean differences or ratios of geometric means with 95% confidence intervals. Odds ratios and 95% confidence intervals will be presented for binary outcomes. Estimates will be obtained from linear (or logistic) regression with adjustment for baseline measurements of the outcomes as well as age and gender in order to improve precision. Clustering will be accounted for by using random effects regression models.

In secondary analyses we will compare effects stratified by gender and then by thirds of deprivation. Here we will focus on marked differences in point estimates. Although we will employ interaction terms to formally investigate differences in effects between these subgroups in the relevant regression models, their power will be limited given the primary research aim (and focus of the sample size calculation) on the main effect of the intervention.

### Economic evaluation

The economic evaluation from a societal perspective will take the form of a cost consequence analysis. This approach is chosen given the number of important primary and secondary outcomes. Resources will be valued as reported by researchers, teachers and parents and using routine data sources. The differences between the two arms in terms of mean cost will be calculated, adjusting for variables and accounting for clustering as in the main analysis and using bias corrected confidence intervals, derived using bootstrapping techniques if necessary. Any methodological or parameter uncertainty will be examined through a series of one way sensitivity analyses.

### Qualitative data analyses

All interviews and focus groups will be audio-taped and transcribed verbatim. All of the transcripts will be read and re-read in order to gain an overall understanding of participants' views and experiences. Data will then be analysed thematically, with NVivo or Atlas software, allowing comparisons to be made within and across the interviews.

### Sample size justification

Our sample size calculation (Table 2) was based on the intracluster correlations (ICCs) for different outcomes and other information collected during pilot/feasibility work, such as distributions of the outcome measurements. For each outcome we examined the number of schools required (assuming 25 pupils per school) to detect at least a 0.25-0.30 standard deviation (SD) difference between pupils in intervention and control schools. Differences of this magnitude have been associated with important public health outcomes [2-4]. Our sample size calculation was driven by our primary outcomes and we have used a two-sided alpha of 5% in these calculations. We also completed sample size calculations for secondary outcomes. Here we used an alpha of 1% to account for multiple testing. We will recruit 60 schools with ~ 1500 pupils, which will provide us with 80-90% power to detect important minimal effects for all primary outcomes and most secondary outcomes (Table 2). The two exceptions are the binary secondary outcomes of overweight/obesity where power is more limited to detect a 20% relative effect but reasonable for a 30% relative effect. All of the sample size calculations allow for a 15% loss to follow-up/missing data, that is, none of the calculations require a number of pupils greater than 1275 (1500 minus 15% (225)).

**Table 2:** Sample size calculations

Outcome	ICC (95%CI)	Alpha (two-sided)	Power	**Minimal effect detectable**^a^	Total number schools (pupils) required
Time spent in MVPA per day^b^	0.07 (0.00, 0.21)	5%	80%	0.25 SD	51 (1274)

Time spent sedentary per day^b^	0.02 (0.00, 0.11)	5%	80%	0.25 SD	51 (1272)

Fruit and vegetable portions per day^c^	0.04 (0.00, 0.09)	5%	85%	0.25 SD	51 (1268)

**Secondary outcomes**					

Self-report time spent screen-viewing	0.00 (0.00, 0.03)	1%	90%	0.25 SD	40 (1000)

Snacks portions per day^c^	0.03 (0.00, 0.07)	1%	90%	0.30 SD	50 (1254)

High fat food portions per day^c^	0.05 (0.00, 0.10)	1%	85%	0.30 SD	51 (1274)

High energy drink portions per day^c^	0.02 (0.00, 0.05)	1%	90%	0.30 SD	43 (1070)

Mean BMI	0.00 (0.00, 0.02)	1%	90%	0.30 SD	32 (794)

Mean Waist	0.05 (0.03, 0.20)	1%	80%	0.30 SD	51 (1274)

% overweight or obese (by BMI)	0.03 (0.01, 0.05)	1%	55%	20%^d^	51 (1274)

% centrally obese (by Waist)	0.06 (0.02, 0.09)	1%	60%	20%^d^	51 (1274)

% overweight or obese (by BMI)	0.03 (0.01, 0.05)	1%	80%	30%^e^	51 (1274)

% centrally obese (by Waist)	0.06 (0.02, 0.09)	1%	82%	30%^e^	51 (1274)

^a ^Difference in mean continuously measured outcomes and relative % difference for binary outcomes; ^b ^Assessed by accelerometer; ^c ^For these outcomes the minimal effect detectable is calculated using SD differences on the log-scale; ^d ^Relative difference (i.e. ability to detect an odds ratio of at least 0.8 or 1.2; ^e ^Relative difference of larger magnitude (i.e. ability to detect an odds ratio of at least 0.7 or 1.3)

SD: standard deviation; Calculations assume similar numbers of schools randomised to control or intervention and ~ 25 pupils per school; In all calculations we have used the ICC upper 95% CI value

### Minimising bias

Randomisation of schools will be concealed from the investigators and research staff by having schools randomised by the Bristol Randomised Trials Collaboration (BRTC). Parents, children and the fieldworkers who collect baseline and follow-up data will not be informed which schools have been randomised to intervention or control arm; nor will other school related staff (other than the teachers). At the teacher training for the intervention schools the importance of not revealing that the school is an intervention school to parents, teachers and other staff who might potentially change behaviour as a result of this knowledge will be emphasised. The qualitative assessment will allow us to explore the extent to which children and parents remembered aspects of the intervention. As noted above, the primary analyses will be completed using intention to treat and we believe loss to follow-up is likely to be small. The process evaluation and audit will help us to understand factors that could have supported the intervention in being effective (should we find it to be so), including lessons being particularly memorable and inspiring, teachers completing all lessons and parents influenced by the interactive homeworks, or conversely why it may not have worked (should that be the case).

### Ethical approval and consent

We have obtained ethical approval from the University of Bristol Faculty of Medicine and Dentistry Committee for Ethics (reference number 101115).

Once schools have agreed to participate in the study, parents/guardians of children in Year 4 will be sent a letter and information sheet about the study with the request to reply with an opt-out consent form for their child for each of the measurements (that is, parents have the opportunity to opt their child out of none, one or more, or all of the individual assessments). Parents/guardians will also be given the opportunity to contact the research team to discuss the study and information about being able to withdraw at any stage. The 'opt-out' method of parental permission has been found to be an ethical and appropriate way of informing parents/guardians of such 'low-risk' prevention research, and to avoid the problems of low response rates and significant sampling bias encountered in research which has used active consent procedures with parents/guardians of young people involved in school-based research [38,39]. In addition, opt-out consent has been chosen because: (a) this was used for the pilot/feasibility studies of AFLY5; and (b) this is consistent with the consent process currently used for measurements in Year 6 school pupils for 'The National Child Measurement Programme' which is administered by the NHS in schools, and involves children having their height and weight measured [40].

An information sheet for the child will be sent at the same time that the letter is sent to the parents. The children will also be given a second copy of this information sheet at the time that measurements are undertaken and at this time they will be asked to give signed assent to each of the measurements. Children will only take part in the measurements for which they provide assent, even if we have parental opt-out consent, i.e. measurements will only be taken when there is both opt-out parental consent and child assent. A similar process of parental opt-out consent and child consent will be used for the child focus groups. Signed written parental and teacher consent to participate in the qualitative interviews will be obtained.

All personal information, including school names, pupil and parent names and contact details, will be stored electronically and in paper form in a secure (password protected/securely locked filing cabinet) way and with limited access by the project administrator and manager. Information on the children's measurements will not be disclosed to teachers, National Health Service organisations or anyone else.

## Discussion

This paper describes the protocol for a cluster RCT (AFLY5) that aims to evaluate a school-based intervention aimed at increasing physical activity, decreasing sedentary behaviour and increasing fruit and vegetable consumption in children aged 9-10 years.

AFLY5 addresses weaknesses in previous school-based trials by using objective assessments of physical activity and sedentary behaviour, rather than relying on child or parent report and by examining outcomes both immediately after the end of the intervention and also 12 months later. These are important as child- or parent-report of activity may bias results towards suggesting a greater effect of the intervention than is true[8] and short-term effects may be a direct result of school-based (compulsory) physical activity and may not translate into long-term changes in behaviour. Our current funding only allows for assessment immediately after and then at 12 months post intervention. Furthermore, after this 12 month long-term follow-up assessment the study participants will move to secondary school. However, we plan to collect data from the parents and children about the secondary school that the child intends to attend, and depending upon the results of the immediate and 12 months follow-up assessments and process evaluation, we may subsequently apply for further funds to extend the follow-up period to assess longer term effects.

AFLY5 will be one of just a small number of such trials that have been conducted in the UK. For intervention studies of complex behaviours that are context specific results may not be generalisable from one country to another. From our search of the published literature and of RCT registers (to identify on-going but not yet published trials) there are 3 published school-based RCTs that have been conducted in the UK,[41-44] and one that is currently registered, and recently started, that we are aware of. These are summarised in Table 3. The BEACHeS trial that started in September 2010 differs in its intervention and its target population (South Asian children) from our proposed RCT. Nonetheless, we would hope to collaborate with the investigators of that study once both are completed in order to provide a more comprehensive understanding of how healthy activity and dietary behaviours may be effectively promoted in UK school children. Whilst we acknowledge that interventions might not generalise from one country to another our intervention is based on an original US intervention[15] that we successfully modified for use in the UK,[13,14] and we hope that our process evaluation in AFLY5 will provide sufficient information to make it generalisable to a wide range of populations should it be shown to be effective.

**Table 3:** School-based intervention studies in the UK

Study	Design N Schools (Children)	Participants & setting	Intervention	Key results
Be Smart [41]	Non-cluster pilot RCT 3 (213)	Age: 5-7; Area: Oxford	3 interventions with lessons to: 1. Promote healthy eating 2. Promote physical activity 3. Both 1 & 2	Improved knowledge and intake of fruit and vegetables with all interventions. No effect on BMI

APPLES [42]	Cluster RCT 10 (634)	Age: 7-11; Area: Leeds	Teacher training to promote healthy lifestyle; modification of school dinners & tuck shop; increased PE classes and playground activities	Modest increase in vegetable consumption. No effect on sedentary behaviour, physical activity, BMI

CHOPPS [43,44]	Cluster RCT 6 (644)	Age: 7-11; Area: Dorset	Focused lesson based education programme aimed at discouraging the consumption of carbonated drinks	Mean difference in carbonated drinks comparing pupils from control to intervention schools: 0.7 glasses/3 days (95%CI:0.1, 1.3); mean difference in % overweight 7.7% (2.2, 13.1%) Recent long-term follow-up (2 years after end of intervention): Mean difference in change in BMI z-score 0.10 (95%:0.00, 0.21) and in % overweight 4.6%(-4.3, 13.5%) comparing control to intervention

BEACHeS	Cluster RCT Registered and was due to start 1/9/2010. Target sample 44 (1922)	Age 9-10; Schools with predominantly or large south Asian population Area: Birmingham	Increase curricular based activity levels; health cooking sessions with parents; interaction with local football team (Aston Villa); information about local leisure facilities	N/A - trial currently underway

Any amendments or updates to this protocol will be lodged with the journal in a way that links them to this protocol document so that all future assessment of the trial publications and conclusions will be able to assess the extent to which we have adhered to the protocol.

### Current (17^th ^July 2011) study status

We have obtained ethics approval and funding for the AFLY5 study and have recruited the programme manager, administrator and fieldworkers. Invitations to participate were sent to the heads of the 153 eligible schools covered by Bristol City (N = 93 eligible schools) and North Somerset (N = 60 eligible schools) councils between 17^th ^February and 3^rd ^May 2011 and by early July we had successfully recruited 60 schools. In addition to these 60 schools one consented to participate but we realised that the school was not eligible as they were already using some of the intervention study material. Thirty schools responded and said that they would not participate, the majority of these said that they were interested but with other commitments could not take part in this study. To date, 62 schools have not responded.

The 60 schools that have consented to take part include ~2500 eligible pupils - i.e. the school year sizes are considerably larger than we anticipated form our pilot work. We have decided to include all 60 schools and invite all eligible pupils from these schools.

Baseline assessments have been completed on pupils from 31 of the recruited schools to date.

## List of abbreviations

AFLY5: Active For Life Year 5; BMI: Body Mass Index; CI: Confidence Interval; ICC: Intracluster correlation coefficient; MVPA: Moderate or Vigorous Physical Activity; PA: Physical Activity; PE: Physical Education; RCT: Randomised Controlled Trial; SD: Standard Deviation; TV: Television; UK: United Kingdom; WC: Waist Circumference.

## Competing interests

The authors declare that they have no competing interests.

## Authors' contributions

DAL, RJ and RK completed all phases of pilot and feasibility study for AFLY5; DAL wrote the first draft of this paper, completed sample size calculations and developed the quantitative analysis protocol; RJ and RK developed the qualitative methods and analysis protocol for data collected from these; SMN developed the economic evaluation methods and associated analysis protocol; TJP provided advice on the sample size calculations and the quantitative analysis protocol; all authors contributed to the final version of the paper and will be responsible for conducting the AFLY5 study.
